# Incidence and Geographical Variation of Amyotrophic Lateral Sclerosis (ALS) in Southern Germany – Completeness of the ALS Registry Swabia

**DOI:** 10.1371/journal.pone.0093932

**Published:** 2014-04-10

**Authors:** Hatice Uenal, Angela Rosenbohm, Johannes Kufeldt, Patrick Weydt, Katharina Goder, Albert Ludolph, Dietrich Rothenbacher, Gabriele Nagel, and the ALS registry Study Group

**Affiliations:** 1 Institute for Epidemiology and Medical Biometry, Ulm University, Ulm, Germany; 2 Department of Neurology, Ulm University Clinic, Ulm, Germany; Inserm, France

## Abstract

Objective of this paper was to investigate the incidence, potential geographical clusters and the completeness of the amyotrophic lateral sclerosis (ALS) registry in Southern Germany (Swabia).

Age-standardized incidence rates (ASR) and ratios (SIR) as well as 95% confidence intervals (CI) were estimated at county level. Capture-recapture (CARE) procedures were applied taking data source dependency into account to estimate the quality of case ascertainment in the ALS registry Swabia. We identified 438 ALS cases (53% men, 47% women) in the target population of about 8.4 Mio inhabitants. The gender ratio (men∶women) was 1.1∶1. The mean age at onset of ALS was 63.8 (SD = 11.9) years for men and 66.0 (12.2) for women. The age distribution peaked in the age group 70–74 years. The ASR of ALS was 2.5 per 100,000 person years (PY; 95% CI: 2.3–2.7). The mean SIR was 1.1 per 100,000 PY (95% CI: 1.0–1.2). High SIR suggesting geographical clusters were observed in two counties (Göppingen and Bodenseekreis), but the variation was not statistically significant (p-values = 0.2 and 0.5). The percentage of CARE estimated missing cases was 18.9% in the registry yielding an ASR of 3.1 per 100,000 PY. The high coverage of the CARE estimated completeness of the ALS registry Swabia indicates excellent quality for future projects. Regional variations have to be investigated further.

## Introduction

### Background

In Germany, ALS data on population-based incidence is lacking. The incidence of ALS is estimated to be 2–3 cases per 100,000 person years (PY) in European countries [1]–[3]. During the past years, a rise of the incidence of ALS has been discussed in countries such as France, Sweden and Finland [4]–[6]. The age distribution is reported to peak in the age group 70–74 years [3], [7], [8]. The ratio males/females can be as high as 2.6∶1 [2] but recent studies report a more balanced gender ratio [9], [10].

Very little is known about etiological risk factors of ALS. About 10% of all ALS cases show a clearly Mendelian inheritance pattern. The remainder is isolated in nature and termed sporadic. Previous studies from Europe and the Americas investigating possible environmental influences, reported significant geographical clusters [11]–[15]. The identification of such variation may be an important starting point to pinpoint potential environmental and other risk factors. An important prerequisite for the analysis of registry data is the knowledge of the completeness of the ALS cases in the target population.

ALS registries have been implemented in different regions and case ascertainment is typically estimated through information from multiple sources. However, especially in rare diseases, completeness of case ascertainment is crucial to obtain valid estimates and poses a special challenge. A recent US-based study, estimated missing almost 10% of ALS cases [16]. In European countries, capture-recapture (CARE) identified 19.6% missing cases in a 4 years period were estimated in the Netherlands. [7] and 34.7% CARE missing cases in the Limousin region of France [17].

### Objectives

The main objectives of this paper are firstly, to explore the incidence of ALS and investigate potential geographical variation and secondly, to estimate the completeness of the ALS registry in the region of Swabia (Southern Germany), by means of capture-recapture methods.

## Materials and Methods

### ALS registry Swabia

#### Ethics Statement

International, national and state rules were followed implementing the ALS registry Swabia. We obtained full ethical approval of the ethical committees of Ulm University and the regional medical associations (Landesärztekammer Baden-Württemberg and Landesärztekammer Bayern).

Regional cooperation partners identified ALS patients and obtained written informed consent, before notifying the coordination center at Ulm University. From patients who deceased before the home visit for data collection took place, a minimal data set (3 variables: zip code, age (not date of birth) and gender) was registered anonymously and included in the analysis after duplication of record had been excluded.

#### Study design and study population

The ALS registry Swabia is a clinical-epidemiological registry with the aim to collect data on all newly diagnosed ALS cases in the target population, to estimate epidemiological parameters such as incidence and to describe patient- and disease characteristics, such as natural history of ALS. We have described the registry in more detail elsewhere [18], [19]. The study population consists of all inhabitants living in the region of Swabia (approximately 8.4 million in 2008 and 2009). The exact study region is defined by city and county borders and largely reflects the geographical region of Swabia.

#### Registry cases (inclusion and exclusion criteria)

The inclusion and exclusion criteria for the ALS registry are defined by the diagnosis of possible, probable or definite ALS according to the revised El Escorial criteria for international comparison with epidemiological ALS registries [19]–[22]. Date of diagnosis and residence at the time of diagnosis were recorded. Cases comprise the International Classification of Diseases (ICD) -10 code G.12.2. Regional cooperation partners identified potential patients and obtained informed consent before they notified the ALS registry at Ulm University. In order to achieve maximal standardization, all cases are reviewed by an experienced neurologist according to standardized criteria

#### Study design

The ALS registry is an observational, population based cohort study with a retrospective (all newly diagnosed ALS cases between October 01, 2008 – September 30, 2010) and prospective (newly diagnosed ALS cases as from October 01, 2010) arm. Due to the well-distributed network of neurological centers in the region of Swabia, the ALS patients in Southern Germany are comprehensively recorded.

In the present study, we included only retrospective ALS data registered until end of December 2012. The retrospective branch provided first estimates of the incidence and geographical variation of ALS cases. This enabled us to explore the completeness of the registry by means of capture-recapture case ascertainment. Due to the retrospective data collection, a diagnosis of ALS was most likely.

#### Data collection and quality assurance

Details can be found in previous publications [18], [23]. In brief, all new suspected ALS cases are reported to the data collection office of the ALS registry Swabia at the Department of Neurology of Ulm University. The reports are checked for eligibility for inclusion and thereafter the encryption of personal data is performed. The data coordinating and analysis center is located at the Institute of Epidemiology and Medical Biometry of Ulm University. Here only pseudonymized data of the cases are stored and internal quality checks and analyses are performed on a routine basis.

#### Epidemiological measures and county levels

We calculated the age-standardized rates (ASR) for the target population per 100,000 PY based on the exact European population of the year 2010 with a 95% confidence interval (CI). We applied the direct method in 5-year age-categories [24] over all counties combined as well as for each county in Southern Germany. To determine possible geographical clusters, we displayed small-scale estimated epidemiological measures at county-level. To compare the influence of the different population weights and achieve comparability with other published data, the ASRs were additionally calculated with the European standard population of the year 1990 and an old European standard population of the year 1976 [25]. To visualize counties in which significantly more ALS cases were observed than expected, we also determined age-standardized incidence ratios (SIR) at county-level. Expected ALS cases in each county were estimated using 5-years age class rates of combined European data from 6 ALS registries [3]. Analysis for this paper was performed using SAS software [26]. Increased rates of counties were investigated with Spatial Scan Statistics in SatScan [27].

#### Completeness of the registry - capture-recapture estimation methods

For the statistical CARE procedures, we included five ALS data sources with all natural registered ALS cases in the respective ALS data source, which were according to the health care characteristics of the notifying partners:

(1) ALS registry Swabia, (2) clinical centers, (3) university clinics, (4) small clinics and hospitals, (5) private neurological practice doctors and medical care centers.

The information of ALS cases in the respective data source intersections (multiple records) is the main precondition in CARE methods. The simplest method is the origin variant of Petersen dual system estimator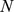, a simple equation of 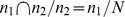 [28]. It is commonly used in the wildness to track animal populations with only 2 sources, where animals were captured and marked in a population (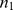) and recaptured again (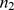).

We compared several statistical CARE procedures: simple Petersen estimates with simple techniques of the set theory [28], Chapman estimates [29], nearly unbiased maximum likelihood estimator modified firstly by Schnabel [29]–[31], Schnabel/Chapman estimates for Poisson distributions for the sparse data in rare diseases [32], and log linear model approaches to control for bias [33]. With regard to the latter, possible dependence between data sources is taken under consideration with log linear approaches to adjust among multiple sources. This method requires that data source characteristics (including intersections) of each patient are separately modeled for the main log linear model.

According to the requirements of the linear model, we categorized our ALS cases by their natural occurrence in the data source intersections and applied the interactive freeware program CARE [34] in combination with Gsrun 5.0 according to the suggestions of Chao et al. [33] to ascertain possible missing cases in the ALS registry Swabia.

## Results

### ALS registry Swabia (retrospective ALS cases)

In total, 520 suspected ALS cases were reported, of whom 8 cases could not be confirmed, and 74 ALS cases were multiple recorded. Thus, 438 confirmed ALS cases remained, of whom 53% were men and 47% women. The gender ratio (men∶women) was 1.1. The age distribution of all ALS cases peaked in the age group 70–74 years. Men peaked in the age group 65–69 and women in 70–74 age group. Male patients had a mean age at diagnosis (SD) of 63.8 (11.9) years and female patients a mean age at diagnosis of 66.0 (12.2) years.

### Epidemiological measures and results on county levels

Overall, the crude incidence was estimated to be 2.6 per 100,000 PY. The age-standardized exact European population (data from year 2010) incidence rate was 2.5 per 100,000 PY (95% CI: 2.3–2.7). The 95% CI based on the Poisson distribution did not differ.

**Figure 1:**
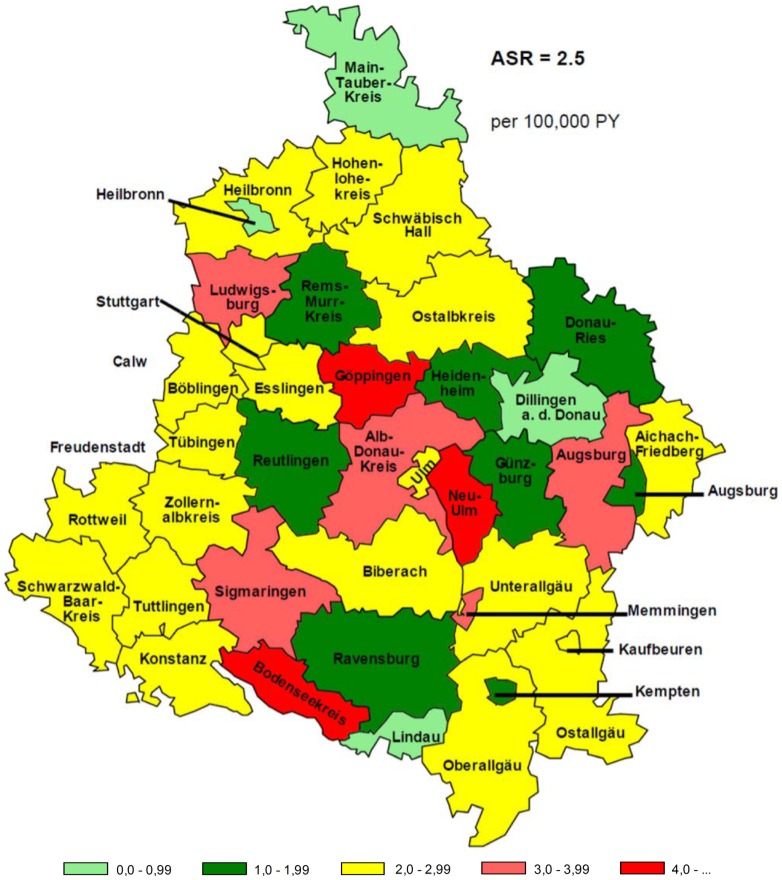
Comparison of age standardized incidence rates of ALS registry data at county level with the exact European populations.

Depending on the selection of the European population to standardize to, the ASRs for retrospective data varied from 1.9 to 2.5 per 100,000 PY for the whole study region and differed in their geographical representation of the 40 counties. A comparison of ASRs at county level is given in **Fig. 1** **.**

As displayed in **Fig. 1**, using the exact EU population, the highest ASR was observed in the county Bodenseekreis with 4.4 per 100,000 PY and the lowest with 0.6 per 100,000 PY in the county Lindau. With other EU standard populations, other high incidence counties disappeared (e.g. Göppingen with the old standard population of 1976).

Spatial Scan statistics and hypothesis testing were therefore considered to test for geographical differences.

**Figure 2:**
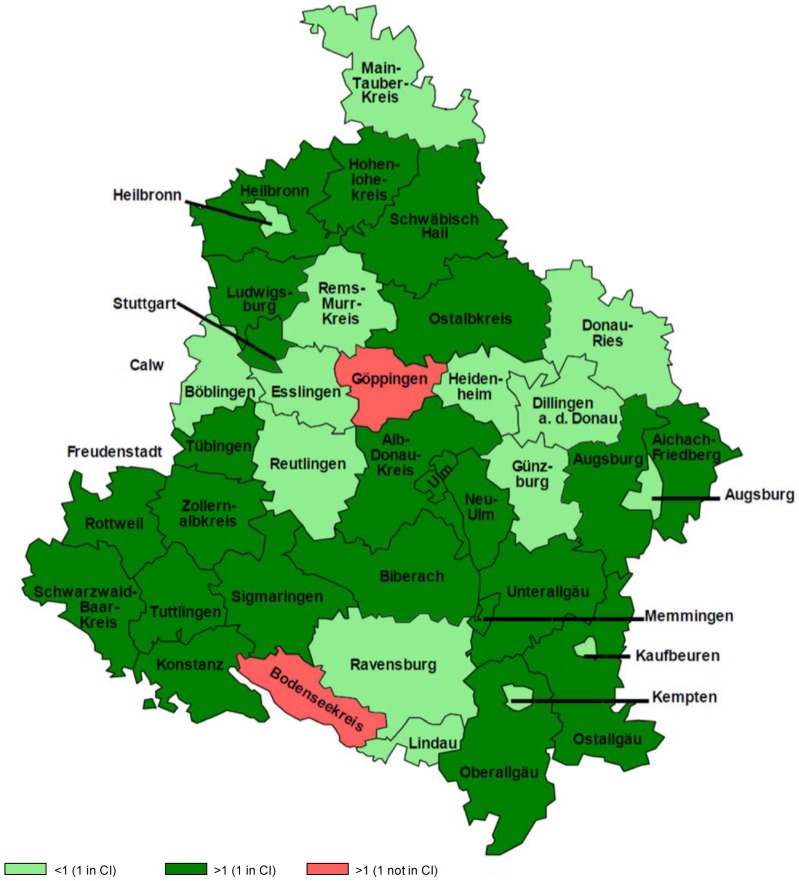
Age standardized incidence ratios of ALS registry data at county level. **Abbreviation and explanation:** significant counties (light green: ‘Confidence Interval’ CI below the cut-off value of 1 means less observed cases as expected; dark red: CI above the cut-off value means more observed cases as expected), not significant counties are displayed in dark-green and light red.

Comparing the 40 counties in the target population, high-ratio counties (Göppingen: SIR = 1.9, CI = 1.2–2.8; ‘Bodensee’: SIR = 1.8, CI = 1.1–2.8; **Fig. 2** (dark-red colored)) were determined, but the difference in variation in the overall pattern was not statistically significant (p-value = 0.17 and p-value = 0.43, respectively).

### Completeness of the registry - capture-recapture estimation methods

**Figure 3:**
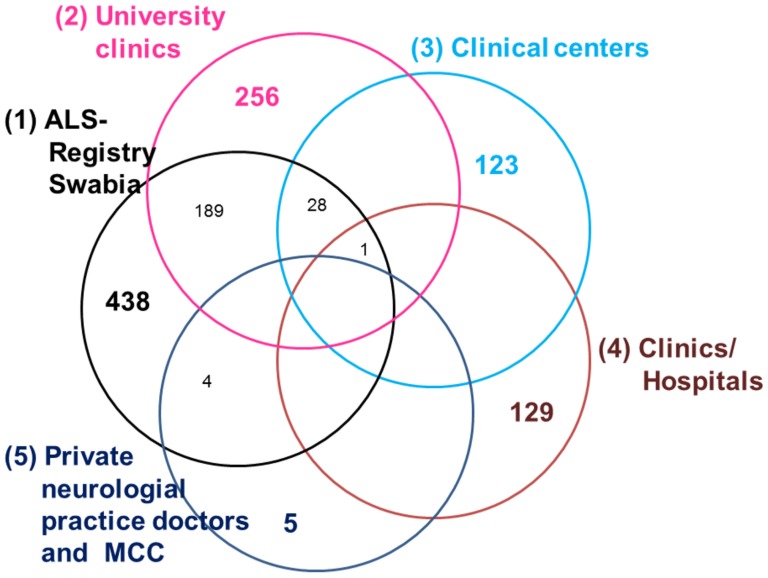
Data source intersections of the ALS cases in Southern Germany. Abbreviation: MCC Medical Care Centers. Other Intersections: (1),(3),(4) = 2; (1),(3) = 91; (1),(4) = 95; (1),(2),(4) = 31;

Due to multiple information of ALS recording, the case ascertainment of the ALS registry Swabia was estimated by 5 data sources:

(1) ALS registry Swabia (N = 438); (2) University clinics (N = 256); (3) clinical centers (N = 122); (4) small clinics and hospitals (N = 130) and (5) private neurological practice doctors and medical care centers (N = 4) **(** **Fig. 3** **)**.

The data source intersections of registered ALS cases to be present or absent are shown in **Fig. 3**.

The percentage of log-linear estimated missing ALS cases in the target population was estimated to be 18.9%. Correspondingly, a maximum total of 540 (SD = 13.1) ALS cases is estimated where source dependency is taken into account and estimation bias were minimized in optimizing logistical approaches. Compared to other statistical CARE procedures, the log-linear model included all available intersections of the data sources and took the dependency between the data sources into account. Alternative methods, which are considered less accurate, yielded results from 15.8% to 35.7% depending on the chosen estimation method and the data sources included (**table 1**).

**Table 1:** Case ascertainment of the ALS cases in Southern Germany compared by different statistical procedures of capture-recapture methods.

CARE – Estimation Method	Case ascertainment:		
	Estimated ALS cases in ALS registry Swabia		
Observed ALS cases:	Missing	CARE	Included interaction terms
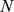 = 438	(%)	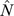	(data sources)
**Log Linear Model**	**18.9**	**539.9**	SD = 13.1
			(1)*(2); (1)*(3); (1)*(4); (1)*(5); (2)*(3); (2)*(4); (2)*(5); (3)*(4); (3)*(5); (4)*(5)
*Alternative approaches:*			
**Peterson Estimates**	21.1	555	(1)*(2); (1)*(3); (1)*(4); (1)*(5)
**Schnabel/Chapman Estimates**	25.3	586	(1)*(2); (1)*(3); (1)*(4); (1)*(5); (2)*(5); (3)*(5); (4)*(5)
	25.6	589	(1)*(2); (1)*(3); (1)*(4)
**Chapman Estimates**	26.0	592	(1)*(2); (1)*(3); (1)*(4); (1)*(5); (2)*(5); (3)*(5); (4)*(5)
	15.8	520	(1)*(2); (1)*(3); (1)*(4); (1)*(5); (3)*(5); (4)*(5)
**Schnabel modificated Estimates**	35.7	681	(1)*(2); (1)*(3); (1)*(4); (1)*(5); (2)*(5); (3)*(5); (4)*(5)
	24.5	580	(1)*(2); (1)*(3); (1)*(4); (1)*(5); (3)*(5); (4)*(5)

**Abbreviations:** 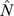 estimated total number; SD Standard Deviation; CARE capture-recapture Methods.

In our study, the alternative CARE methods turned out to be conservative in source dependency in relation to sparse data and the corresponding results seemed strongly biased depending on the selection of intersections terms. Information of included interaction terms are set out in **table 1**. A comparison of the total estimated ALS cases in the target population (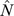 = 520–681) is summarized in **table 1** by the missing percentages regarding the observed total ALS cases (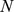 = 438).

Adding the estimated 18.9% CARE missing cases to the observed ALS cases, the ASR increased to 3.1 per 100,000 PY as expected for case ascertainment in the ALS registry before study beginning.

Based on log-linear CARE estimated total ALS cases in the study region, the number of ALS cases, demographic characteristics and the completeness in percentage in the respective data sources are given in the following **table 2**.

**Table 2:** Demographic characteristics of the observed ALS cases in the data sources.

	CARE	ALS	University	Clinical	Clinics/	NPD
	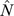	Registry	Clinics	Centers	Hospital	and MCC
Number of cases	**539.9**	438	256	122	130	4
Estimated completeness (*%)*		81%	47%	23%	24%	1%
Age (years): *Mean*		64.2	61.5	64.6	67.6	66.3
*(SD)*		(12.1)	(11.3)	(12.4)	(11.1)	(19.4)
Gender ratio *(men:women)*		1.1	1.4	0.8	0.8	1

**Abbreviations:**

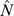
 estimated total number; CARE capture-recapture methods; NPD and MCC: private neurological practice doctors and medical care centers.

## Discussion

### Summary of the main results

Based on the first population based ALS registry in Germany covering a geographically defined region with about 8.4 million inhabitants, a crude incidence rate of ALS of 2.6 per 100,000 PY was estimated. The exact European ASR was 2.5 per 100,000 PY (95%-CI: 2.3 to 2.7). The male/female ratio was 1.1 and the age-distribution of the ALS cases peaked in the age group 70–74 years. The observed incidence rates and the peak were consistent with findings from other European countries, indicating that the ALS registry Swabia is well implemented. Although we found some geographical incidence variation, the differences were not statistically significant and most likely represent chance variation. The estimated completeness was 81.1% and indicates a good coverage of the registry. After correction for potentially missing cases, the ASR was estimated to a maximum of 3.1 per 100,000 PY.

### Epidemiological measures of ALS

In our study, the crude annual incidence rate for 2009/2010 was comparable to the estimated average crude incidence rate of ALS in Europe. The ASR in Swabia was in line with the different ASRs ranging in European countries from a low of 1.5 in Lancashire and a high of 2.7 in Ireland per 100,000 PY [3], [35]. A peak incidence in the age group 70–74 years is also reported by other authors [3], [7], [8]. A decreasing trend in the gender ratio towards one as described in recent studies [9], [10] was clearly evident from our data. Our study results were also in line with ALS-incidence rates in Canada, which range from 1.6 to 2.4 for crude incidences and 2.1 to 2.3 for ASRs [36]. Higher ASRs were observed in previous studies (USA standard population) for the age group of 45 to 74 years in studies from Canada, Denmark, Finland, Italy, Ireland, Israel, Sardinia, Scotland, Sweden, USA, with a ASR range of 2.1 (Israel) to 8.5 (Middle Finland) [8].

These differences can be explained e.g. by to possible geographical variation, the underlying population size, the completeness of the registries or bias produced by weights of the standard population. Wide 95% CIs of ASRs could also partly explain the variation in the ASRs, possibly revealing different methods used in the registries to estimate epidemiological parameters. Analyzing ALS data, the geographical variation of incidences might be a result of the standard deviation or standard error occurring from the issue of sparse data. In 1996, Pringle mentioned that a compromise could be summarizing two extremes, small scaled cartographical presentation of the estimated risks (focus attention on smaller areas) and probability maps (focus attention on larger areas). Empirical Bayesian approaches offer such a possible solution by providing estimates of relative risks e.g. for each county and converting them into rates by simply multiplying them by the overall rate [37]. Future studies that compare Bayesian corrected epidemiological measures at county level with the measures estimated in this work, should be considered since enhanced ALS data are provided and maybe added for underreported counties. To reduce bias, additional estimates for the new, old and exact European standard population were calculated in our study with an ASR of 2.0, 1.9 and 2.5 respectively. Based on the SIRs ascertained with the suggested European rates in [3], more ALS cases were expected in some counties suggesting underreporting in these counties (light green colored; **Fig. 2**). Possible explanations for rates lower than expected might be that patients corresponding to the region visited clinics in neighboring counties. However, all clinics in the study region as well as clinics in neighboring counties outside the study region were contacted at regular intervals (3 to 6 months) to reduce delayed notifications and to improve the data acquisition. Counties with high SIR are suggestive for disease clusters and could be related to familiar cases. However, the familial cases were not observed in the two counties with high SIR in our study. Thus, possible other risk factors or as yet unidentified familial cases should be explored in future studies.

The completeness of the registry might also be influenced by unknown or delayed diagnosis. Generally, previous studies reported a median of about 9–13 months diagnostic delay from symptom onset and a possible underreporting in some geographic areas [3], [38]–[40].

### Comparison with the literature on completeness of ALS registries - capture-recapture estimation methods

The estimated percentage of CARE missing ALS cases in our study (range of 15.8% to 35.7%) has to be compared with other ALS registries from a low of 2.2% in Scotland [41] or Uruguay of 3.5% [14] and a high of 31.2% in Texas (Harris County) [28] or in France (Limousin) of 34.7% [17]. Consistent with our findings, a study from the Netherlands [7] reported with 19.6% CARE missing ALS cases and a CARE estimated incidence rate of 2.8 in a study population of 16.5 Mio inhabitants. However, differences could be explained by the different regions with different conditions or due to different methods used. Differences in the number of data sources included, the interaction terms the study period chosen or the data source dependency considered in a model, were all discussed as potential sources of bias [7], [16], [17], [28], [42].

In terms of the estimation of the registry completeness, several statistical procedures with CARE methods were compared to consider the sparse data in each data source and to reduce estimation bias caused by methodological approaches (**Methods S1**). Although the models (especially Poisson distribution related) seem to be robust concerning sparse data, results show large variation as soon as more data sources are excluded from the model. A robust model for example was presented by Schnabel in Chapman et al. [32], with the advantage of each intersection of the data sources being approximately Poisson distributed [32]. However, main limitations with CARE methods are the limited accuracy of estimate due to uncertainties in source dependencies. The application of these methods was complex to evaluate the completeness of the registry. The case ascertainment was evaluated partly in some of these methods strongly biased regarding the intersection terms in our study as well as CARE estimates with only 2 data sources due to the source dependencies. Similar observations were made in the ALS study of Wittie et al. [16], [17], [28]. We therefore compared log-linear approaches to take the source dependency into account, where each ALS cases had to be modeled as ‘present’ or ‘absent’ in a data source and provided in a separate dataset for the log linear model calculations as suggested by Chao et al. [33]. This method seemed to be the most adequate method in estimating the case ascertainment of rare diseases, because the dependency between multiple data sources of sparse data are included as separate log functions into the main model [33].

### Strength and limitations

In general, our results were comparable to data on epidemiological characteristics collected in the EURALS project [43], [44], which facilitated the data quality evaluation. Data quality assurance and plausibility-check procedures have been implemented ensuring high standards of our data collection and management procedures. Newly diagnosed ALS cases were reviewed by experienced neurologists according to the revised EL Escorial criteria [20]–[22] to assure high international standards.

To maintain a high completeness of the registry, constant professional training of study staff and collaborators and the availability of study nurses to recover all ALS cases all over the study region as was provided. Due to the diagnosis delay, data on retrospective patients were still recorded long after the end of the recruitment period in the retrospective arm of the study. Another limitation was the lack of access to an independent mortality data source on an individual level, due to German administrative regulations.

### Outlook

The high coverage of the CARE estimated completeness of the successfully implemented ALS registry Swabia indicates excellent quality for future projects. Interesting research aspects will cover a cluster analysis at zip code level by means of spatial scan statistics including potential, environmental risk factors in ALS cases at zip code level.

Following cooperating partners provided data for the ALS registry Swabia:

## Supporting Information

Methods S1 (PDF)
