# Supplementary material for: Incidence and Geographical Variation of Amyotrophic Lateral Sclerosis (ALS) in Southern Germany – Completeness of the ALS Registry Swabia
Source: PLoS One. 2014 Apr 10;9(4):e93932. doi: 10.1371/journal.pone.0093932 (PMC3983245; doi:10.1371/journal.pone.0093932)
Supplement: Methods S1 — (PDF) [file pone.0093932.s001.pdf]

Methods S1 Supplement: Comparison of Capture-Recapture – methods

We calculated the simple Petersen estimate shown as follows with simple techniques of the set theory as well as Chapman estimates to control for bias adjusting with log linear model approaches and comparing the results. The origin variant, the Petersen dual system estimator $\hat{N}$, is a simple equation of $n_1 \cap n_2 / n_2 = n_1 / N$, where n_1 and n_2 are per 2 selected data sources [27]:

and $$\hat{N} = \frac{(n_1+1)(n_2+1)}{n_1 \cap n_2 + 1} - 1$$

as nearly unbiased maximum likelihood estimator for Poisson-distributions modified firstly by Schnabel and Chapman [28, 30, 31].

Huisman et al. [7] calculated in 2011 their complete case ascertainment to detect missing ALS cases with the formula developed by Chapman in [28, 31].

Forbes et al. [40] demonstrated a maximum likelihood estimator in 2007 with ALS cases with 2 data sources, which determined ALS cases present in a unique source are multiplied with ALS cases not present in the source divided by these cases being common in both sources.

In our study, we applied a slight modification of the estimator given by Schnabel [30] for 5 data sources, where it is seen most useful since each $n_{1_i} \cap n_{2_i}$ has approximately a Poisson distribution [31]:

$$N_P = \frac{\sum_{i=1}^{k} n_{1_i} n_{2_i}}{\sum_{i=1}^{k} n_{1_i} \cap n_{2_i} + 1}$$

As a second scenario, we compared the results calculated by the Chapman estimator [27, 32]:

$$N = \sum_{i=1}^{k} \frac{n_{1_i} n_{2_i}}{n_{1_i} \cap n_{2_i} + 1}$$

Possible dependence between data sources is taken under consideration with log linear approaches to adjust among multiple sources. In log-linear modeling, the best model estimating the relation between the ALS data sources was assessed [32, 33].

After determining our ALS patients into the categories of Z_{ijklm} (being present or absent in the 5 data sources; $i, j, k, l, m = 1$ or $i, j, k, l, m = 0$), we applied the interactive freeware program CARE [33] in combination with Gsrun 5.0 according to Chao et al. [32] to ascertain possible missing cases in ALS registry Swabia.
